# A First Generation Microsatellite- and SNP-Based Linkage Map of *Jatropha*

**DOI:** 10.1371/journal.pone.0023632

**Published:** 2011-08-25

**Authors:** Chun Ming Wang, Peng Liu, Chengxin Yi, Keyu Gu, Fei Sun, Lei Li, Loong Chueng Lo, Xiaokun Liu, Felicia Feng, Grace Lin, Suying Cao, Yan Hong, Zhongchao Yin, Gen Hua Yue

**Affiliations:** 1 Molecular Population Genetics Group, Temasek Life Sciences Laboratory, National University of Singapore, Singapore, Singapore; 2 Joil Pte Ltd, Singapore, Singapore; 3 Molecular Plant Pathology Group, Temasek Life Sciences Laboratory, National University of Singapore, Singapore, Singapore; 4 Plant Biotechnology Group, Temasek Life Sciences Laboratory, National University of Singapore, Singapore, Singapore; Rutgers University, United States of America

## Abstract

*Jatropha curcas* is a potential plant species for biodiesel production. However, its seed yield is too low for profitable production of biodiesel. To improve the productivity, genetic improvement through breeding is essential. A linkage map is an important component in molecular breeding. We established a first-generation linkage map using a mapping panel containing two backcross populations with 93 progeny. We mapped 506 markers (216 microsatellites and 290 SNPs from ESTs) onto 11 linkage groups. The total length of the map was 1440.9 cM with an average marker space of 2.8 cM. Blasting of 222 *Jatropha* ESTs containing polymorphic SSR or SNP markers against EST-databases revealed that 91.0%, 86.5% and 79.2% of *Jatropha* ESTs were homologous to counterparts in castor bean, poplar and *Arabidopsis* respectively. Mapping 192 orthologous markers to the assembled whole genome sequence of *Arabidopsis thaliana* identified 38 syntenic blocks and revealed that small linkage blocks were well conserved, but often shuffled. The first generation linkage map and the data of comparative mapping could lay a solid foundation for QTL mapping of agronomic traits, marker-assisted breeding and cloning genes responsible for phenotypic variation.

## Introduction

The increasing demand for diesel coupled with continuous rise in price of crude oil has forced us to search for an ecologically sustainable alternative energy source [1]. Biodiesel from vegetable oil emerged as a viable alternative, particularly non-edible vegetable oil. *Jatropha curcas* L, also called Physic nut, is a perennial poisonous shrub belonging to the Euphorbiaceae family [2]. This plant originating from Central America has been spread to other tropical and subtropical countries and is mainly grown in Asia and Africa. People claimed that *J. curcas* is resistant to a high degree of drought and does not directly compete with food crops [1], [3]. The generation interval of *J. curcas* was only six months in tropical regions and its genome consists of 11 chromosome pairs [4]. Its seeds contain ca. 30% oil that is usable in a standard diesel engine [5], therefore *J. curcas* is regarded as a promising candidate for producing biodiesel [1], [6]. There is increasing interest in the use of *J. curcas* oil to alleviate the energy crisis [3]. However, *J. curcas* has been an uncultivated wild-species, and until recently little is known about its genetics and genome. Although some genes involved in pathways for biosynthesis of fatty acids and lipids have been cloned recently [4], [7], [8], methods for gene silencing have been established [9], and the genome was sequenced [10]. *J. curcas* has never been domesticated and bred for producing oil in large scale. Detailed selective breeding has not been extensively carried out yet. To make the production of *J. curcas* profitable and sustainable, genetic improvement of oil yield and quality, as well as diseases and pests resistance is demanded.

A genetic linkage map is an essential tool in molecular breeding for genetic improvement [11]. Such a map will facilitate genome mapping, genetic dissection of quantitative trait loci (QTL) and positional cloning of important genes, and provide a scaffold for assembling physical maps and an indispensable tool for functional genomics studies [12], [13]. Linkage maps have been constructed in a number of economically important species [14], [15], [16], [17], [18], [19] using different markers, such as protein polymorphisms, RAPD, RFLP, AFLP, microsatellites (also termed as single sequence repeats: SSRs) and SNPs in reference families. Although, a rather large variety of markers exists, each possessing its own advantages and drawbacks, microsatellites are the most preferred markers for linkage mapping [20]. Recently, single nucleotide polymorphism (SNP) markers have attracted significant attention in creating dense genetic linkage maps and genome-wide association studies [21] because SNPs are the most abundant class of polymorphisms in genomes, and can be genotyped cost-effectively [22].

With the elucidation of genes involved in many biochemical pathways, information generated in the model species *Arabidopsis thaliana* holds enormous potential for application in breeding of other crops [23]. Establishment of syntenic relationships between *A. thaliana* and other crops through comparative mapping would be beneficial for the identification of candidate genes contributing to agronomic traits from corresponding regions in *A. thaliana* and also serve as a resource to generate more markers for fine mapping in syntenic regions of other crops [24].

In order to facilitate genetic improvement of *Jatropha*, we constructed a first-generation linkage map comprising 216 microsatellites and 290 SNPs and spanning 1440.9 cM. We generated a comparative map between *J. curcas* and *A. thaliana* containing 192 marker loci derived from expressed sequence tags (ESTs). This linkage map represents the first linkage map of *Jatropha*, and could provide an indispensable and powerful tool for QTL analysis, gene mapping and marker-assisted selection (MAS) in breeding.

## Materials and Methods

### 1. Mapping populations and DNA isolation

Two *J. curcas* individuals PZM16 and ZS-2 were selected as female parents, which were crossed to an individual *Jatropha integerrima* S001 as male parent. The two hybrid offspring, namely CI7041 and CI7018, were generated. Two BC_1_F_1_ populations each containing over 300 offspring were constructed by backcrosses PZM16 X CI7041 and ZS-2 X CI7018, respectively. From the backcrosses PZM16 X CI7041, 50 offspring were used in the construction of the linkage map, whereas from the backcross ZS-2 X CI7018, 43 individuals were used. The mapping populations and their parental lines were planted under standard growth conditions in an experimental farm of Lim Chu Kang farm, Singapore. Total DNA was isolated from leaves using the DNeasy plant mini kit (QIAGEN, Hilden, Germany).

### 2. Identification of microsatellite and SNP markers

Microsatellites were isolated from microsatellite-enriched libraries. Briefly, two partial genomic DNA libraries enriched for GA repeats were constructed as described previously [25], for *J. curcas* and a hybrid between *J. curcas* and *J. integerrima*, respectively. Repeat-enriched DNA fragments between 400 to 1200 bp in length were cloned into pGEM-T vector (Promega, CA, USA), and transformed into XL-10 blue supercompetent cells (Stratagene, CA, USA). The libraries were arrayed into 96-well plates for bidirectional sequencing on an ABI3730xl DNA sequencer (ABI, CA, USA) using the BigDye V3.0 kit and M13 and M13 reverse primers. Redundant and overlapping sequences were grouped using Sequencher (GeneCodes, MI, USA). Sequences containing GA>7 were subjected to primer design using PrimerSelect (DNASTAR, MA, USA), targeting a product size between 100 and 400 bp.

SNPs were detected in the three parents (PZM16, ZS-2 and S001) of the two mapping populations by direct sequencing of PCR products. Briefly, EST sequences from a cDNA library of *Jatropha* developing seeds (unpublished data from Dr Yin's group) were used to design primers for amplification genomic DNA. ESTs were blasted against assembled whole genome sequences of model plant species (i.e. rice and *Arabidopsis*) in GenBank to indentify potential boundary of exons in each EST. Primers (see Table S1) were designed in exons of each EST sequence using PrimerSelect (DNAstar, WI, USA). Expected length of PCR products was longer than 200 bp, but shorter than 1.2 kb. PCR products were sequenced in both directions using the same primers for amplifying the genomic DNA on the ABI3730xl DNA sequencer (ABI, CA, USA) using the BigDye V3.0 kit. Sequences of three parents generated by each primer pair were aligned using Sequencher and SNPs were identified by visual inspection.

### 3. Genotyping of microsatellites and SNPs

Primers were designed for each unique microsatellite sequence using PrimerSelect (DNAstar, WI, USA). One primer of each pair was labeled with FAM or HEX fluorescent dyes at the 5′ end. The PCR program for microsatellite amplifications on PTC-100 PCR machines (MJ Research, CA, USA) consisted of the following steps: 94°C for 2 min followed by 37 cycles of 94°C for 30 s, 55°C for 30 s and 72°C for 45 s, then a final step of 72°C for 5 min. Each PCR reaction consisted of 1× PCR buffer (Finnzymes, Espoo, Finland) with 1.5 mM MgCl_2_, 200 nM of each PCR primer, 50 µM of each dNTP, 10 ng genomic DNA and one unit of DNA-polymerase (Finnzymes, Espoo, Finland). Products were analyzed using a DNA sequencer ABI3730xl, and genotyping was carried out to determine fragment size against the size standard ROX-500 (Applied Biosystems, CA, USA) with software GeneMapper V3.5 (Applied Biosystems, CA, USA).

SNPs were genotyped using bidirectional sequencing of each individual in the mapping reference populations on the ABI3730xl DNA sequencer (ABI, CA, USA) using the BigDye V3.0 kit and primers used for amplifying the genomic DNA. Sequences of each individual at each SNP locus were aligned, and SNP genotypes were scored using software Sequencer 4.10 (Gene Codes, CA, USA) and visual inspection.

All genotypes at each microsatellite and SNP locus were stored in a database for later two-point and multiple-point analysis.

### 4. Linkage mapping

For linkage studies, two BC_1_F_1_ mapping populations, consisting of three parents and 93 progeny, were genotyped. The genotyping data from the two mapping populations were combined and analyzed simultaneously using software CRIMAP 3.0 to detect linkage and build map [26]. The CRIMAP 3.0 software was chosen because it allows simultaneous analysis of several families. Briefly, the TWOPOINT option of CRIMAP was used to detect pair-wise linkages first. The assignment of markers to linkage groups was performed by clustering markers showing pair-wise LOD scores of 3.0 or more. For multipoint analysis of larger linkage groups we used the option BUILD to select markers to be used as a framework for the continuing ordering of additional markers. The option ALL was used subsequently to incorporate the rest of the markers, one at a time. The FLIPS and FIXED options were used finally for evaluating the statistical support of the proposed order and the distance between markers, respectively. All multipoint distances were calculated using the Kosambi function. Map graphics were drawn with MapMaker software [27].

### 5. Comparative genome analysis

*Jatropha* ESTs containing mapped SSR or SNP markers were compared to the annotation of the original EST-database (http://cassava.psc.riken.jp/blast.pl) to identify potential homologues in castor bean, poplar and *Arabidopsis* with E<1×10^−5^ as a threshold [28]. DNA markers on the *Jatropha* linkage map were blasted against the assembled whole genome sequences of a model plant *A. thaliana* to identify conserved syntenies and construct a comparative map.

### *6. GenBank accession numbers*

Sequence data of newly developed and mapped markers were deposited in the EMBL/GenBank Data Libraries (see accession numbers in Table S1 and S2).

## Results

### 1. Identifying and genotyping of microsatellite and SNP markers

From two partial genomic DNA libraries enriched for CA and GA repeats, 4000 clones were sequenced in both directions. A total of 2680 clones contained repeat DNA sequences of CA>7 and GA>7, yielding 1380 unique sequences with microsatellites. Among the 1380 sequences, 629 had enough flanking regions for primer design. Primers were designed for 317 microsatellites and tested on the three parents of the mapping panel containing two full-sib families to check the informativeness of markers for mapping. A total of 245 microsatellite loci were informative in the mapping panel. 296 SNPs were identified which showed polymorphism among the parental lines of the mapping population. To our surprise, in the two *J. curcas* individuals (i.e. PZM16 and ZS-2), all 245 microsatellites were homozygous, while in the *J. integerrima* individual (S001), 65% of these microsatellites were heterozygous, and the remaining microsatellites were homozygous, but the genotypes of the *J. integerrima* individual were different from these of the two *J. curcas* individuals. Therefore two F_1_ hybrid individuals (i.e. CI7041 and CI7018) were all heterozygous at all 245 microsatellite loci. The B_1_CF_1_ families were highly informative for constructing a linkage map.

### 2. Linkage map

**Figure 1:**
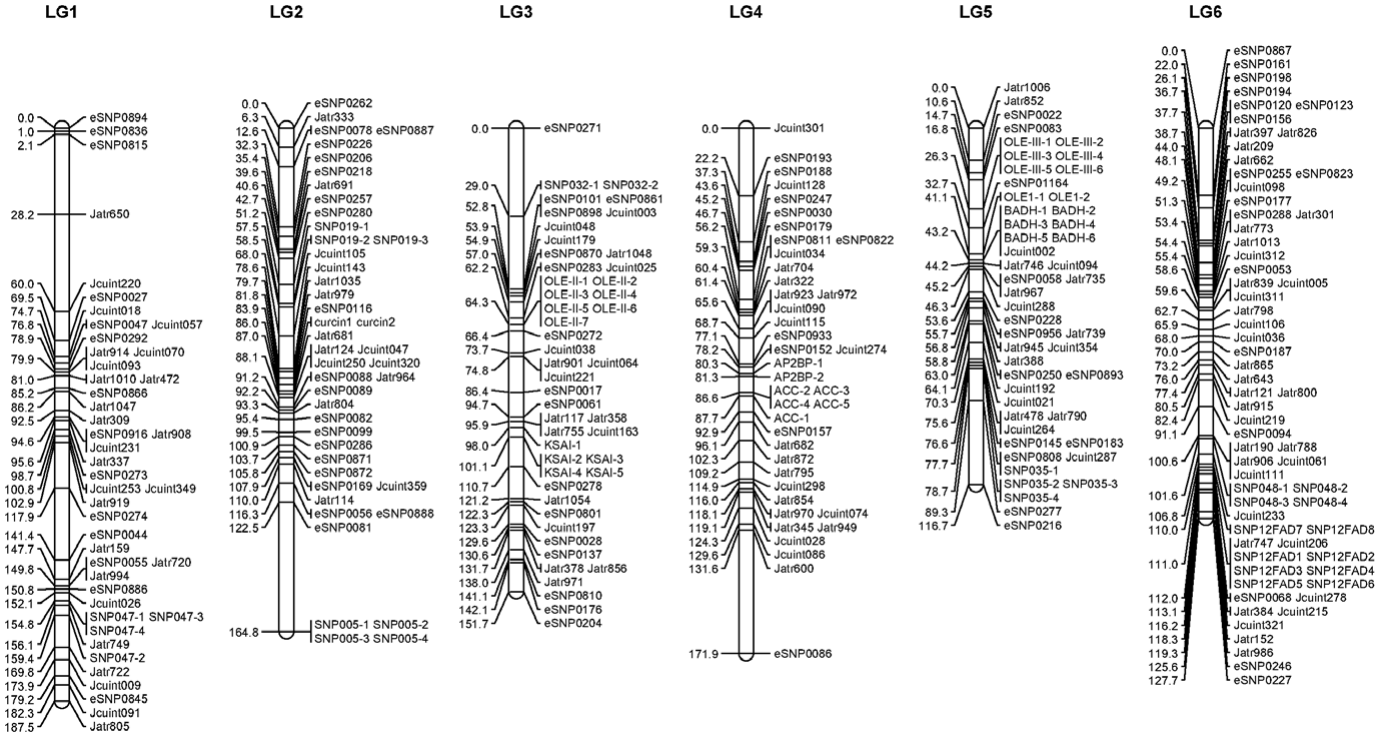
A genetic linkage map of *Jatropha*. Estimates of map distances between markers are indicated in Kosambi centimorgans.

**Figure 2:**
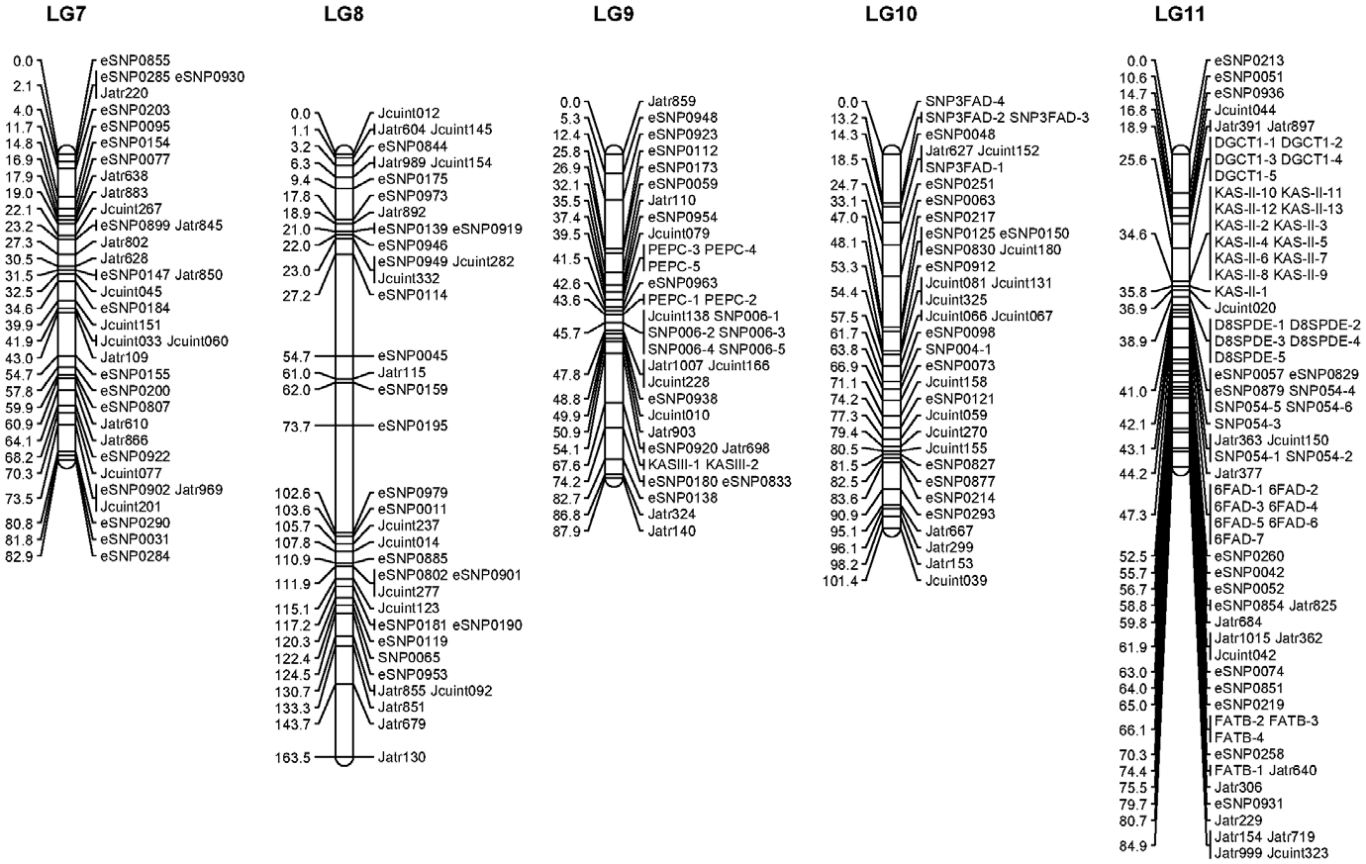
A genetic linkage map of *Jatropha*. Estimates of map distances between markers are indicated in Kosambi centimorgans (continued).

For each of the 245 microsatellite and 296 SNP markers, genotypes were obtained for all 93 offspring. Genotype data of the markers were passed forward into linkage analysis. As a result, 216 microsatellites and 290 SNPs were mapped into 11 linkage groups. Details about primer sequences, PCR product size, and locations of the 216 microsatellites and 290 SNP markers are summarized in Table S1. The remaining 29 informative microsatellites and 6 informative SNPs were not mapped to the linkage map. The length of the 11 linkage groups ranged from 84.9 to 187.5 cM (Table 1 and Fig. 1 and 2). The linkage map covered 1440.9 cM with average marker spacing of 2.8 cM ranging from 1.2 to 4.3 cM. The number of markers on each linkage group ranged from 22 for linkage group 5 (LG5) to 36 for LG6 (see Table 1 and Fig. 1 and 2). LG 11 possessed the highest density of markers with marker spacing shorter than 2 cM, while LGs 1, 4 and 8 had the relatively lower density of markers with marker spacing shorter than 4 cM. The 506 DNA markers were located in 324 discrete positions on 11 linkage groups, therefore, the average space of discrete positions was 4.4 cM ranging from 2.7 for LG 11 to 5.7 cM for LG 1.

**Table 1:** Number of markers and genetic length for each linkage group of the *Jatropha* linkage map.

LG	No. of markers	Unique loci	Length (cM)	cM/marker	cM/unique locus
1	44	33	187.5	4.3	5.7
2	44	33	164.8	3.7	5.0
3	48	28	151.7	3.2	5.4
4	40	30	171.9	4.3	5.7
5	49	22	116.7	2.4	5.3
6	64	36	127.7	2.0	3.5
7	36	31	82.9	2.3	2.7
8	38	29	163.5	4.3	5.6
9	36	24	87.9	2.4	3.7
10	34	27	101.4	3.0	3.8
11	73	31	84.9	1.2	2.7
Total	506	324	1440.9	2.8	4.4

The most spaces between two discrete positions of markers were smaller than 20 cM on the linkage map. However, there were still a few spaces where the distances between two discrete positions of markers were larger than 20 cM, such as eSNP0815-Jatr650-Jcuint220 on LG1, eSNP0081-SNP005-1 on LG2, eSNP0271-SNP032-1 on LG3, Jcuint302-eSNP0193 and Jatr600-eSNP0086 on LG4, eSNP0114-eSNP0045 and eSNP0195-eSNP0979 on LG8. Most of the large spaces were located in the end of the linkage groups.

Segregation ratios that departed from the Mendelian expectation at *P*<0.05 were detected at 80 markers as indicated by asterisks in Table S1. Most of the segregation distortion markers were clustered on LGs 1, 6, 9 and 10. It can be deduced that the suggested segregation distortion loci at these marker regions might link to deleterious alleles. If these loci in the regions are removed from the analysis, <5% of the remaining markers show significant distortion, as expected by chance. Similar phenomena were reported in the linkage maps we constructed for Asian seabass [29].

### 3. Comparative genome analysis

To conduct the comparative genome analysis to identify conserved syntenies, BlastX searches were performed for 222 ESTs containing mapped SSR or SNP markers. Results of the comparative mapping are summarized in Table S2. Highest percentage of the marker sequences of *Jatropha* could be assigned to 215 ESTs (96.8%) in castor bean, followed by poplar 202 (91.0%) (Table S2) whose genomic sequence has been determined.

As searches retrieved 192 (86.5%) sequences from *Arabidopsis*, we compared the *Jatropha* linkage maps to sequence maps of the *Arabidopsis* chromosomes which are available. An uneven distribution of Ath loci originating from each *Arabidopsis* chromosome was observed in the genome of *Jatropha*. Among the 11 LGs of *Jatropha*, all the LGs except 3, 4, 9, 10 and 11 contained Ath loci from each of the five *Arabidopsis* chromosomes (Ath Chr1–Ath Chr5). LGs 3 and 10 were devoid of loci from AthChr2 and AthChr4 respectively. LGs 4, 9 and 11 did not contain any locus from AthChr3 (Table S2).

**Figure 3:**
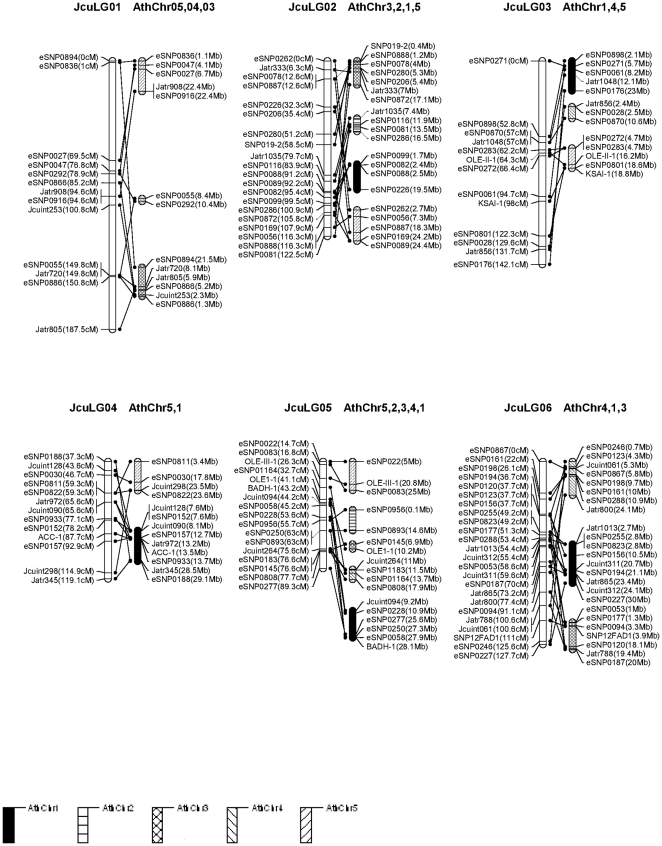
A comparative map between *Jatropha* (Jcu) and *Arabidopsis* (Ath). Orthologous Jcu and Ath chromosomes are shown with lines connecting orthologous markers.

**Figure 4:**
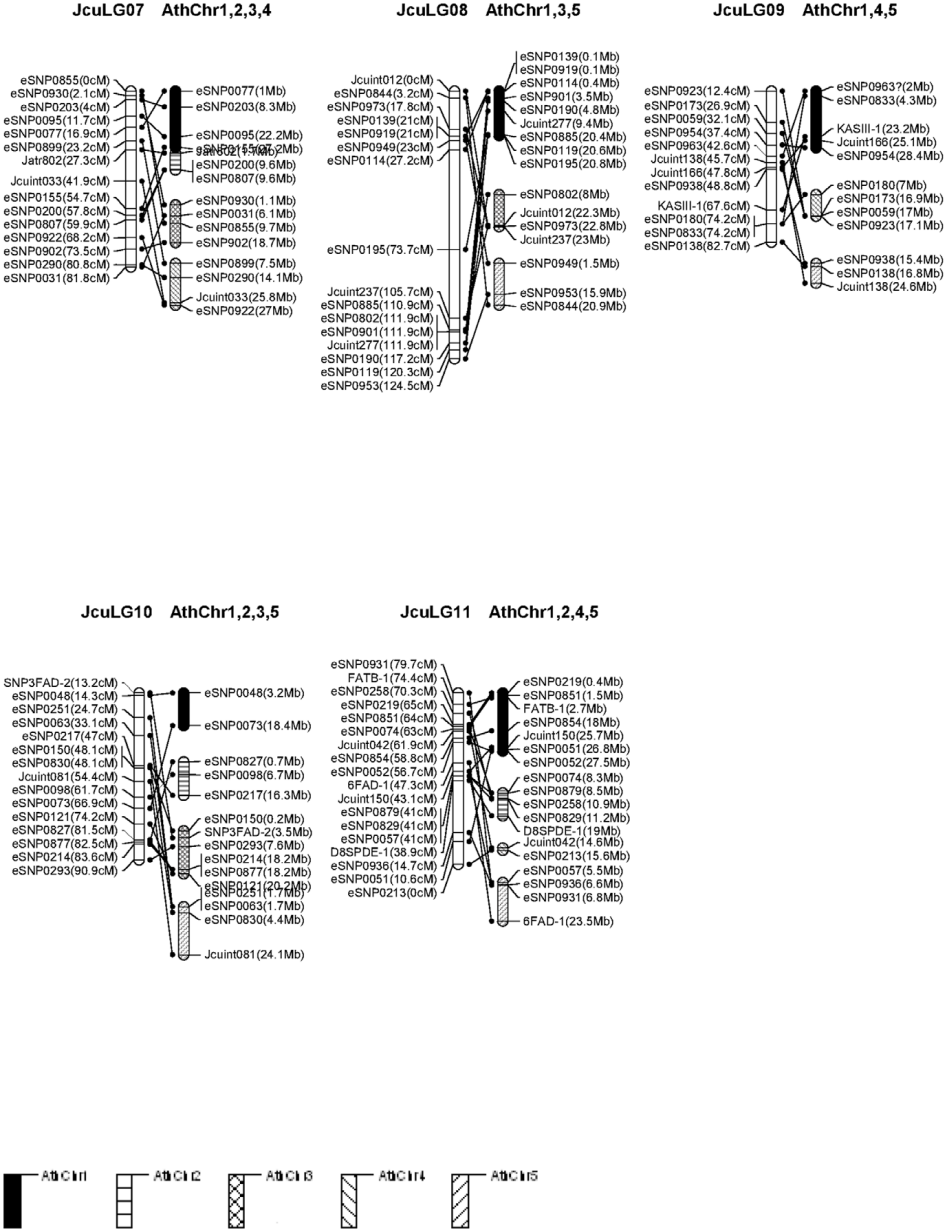
A comparative map between *Jatropha* (Jcu) and *Arabidopsis* (Ath). Orthologous Jcu and Ath chromosomes are shown with lines connecting orthologous markers (continued).

The conserved blocks, which were defined as regions that contained at least two Ath loci from the same block region, were drawn on Fig. 3 and 4. As a result, 176 (79.2%) of the markers were corresponded to sequences mapping to loci in the *Arabidopsis* genome. A total of 38 genomic blocks from *Arabidopsis* genome were identified in the genome of *Jatropha* with an average of 2.8 paralogous blocks for each *Jatropha* linkage group. Fig. 3 and 4 show the comparative map of the 11 *Jatropha* linkage groups and the 5 *Arabidopsis* chromosomes. Conserved synteny blocks were identified in all 11 *Jatropha* linkage groups, each of which contained from 2 to 5 *Arabidopsis* chromosomal blocks. The largest synteny block conserved between *Jatropha* and *Arabidopsis* was found in LG 6 with 24 markers spanning 128.7 cM in the *Jatropha* linkage group and their best matches spanning 3 fragments in chromosomes 1, 3 and 4 of the *Arabidopsis* genome.

## Discussion

### 1. DNA Markers

Although several kinds of genetic markers have been used in the construction of linkage maps [13], [16], [29], [30], due to the quick development of sequencing technologies, sequence-based DNA markers (i.e. microsatellites and SNPs) are preferred markers for linkage mapping. In this study we have developed microsatellites and SNPs for constructing a linkage map of *Jatropha*. The first batch of 245 microsatellites and 296 SNPs was applied to construct the first generation linkage map. The remaining microsatellites will be genotyped and incorporated into the linkage map to increase marker density for QTL analysis for important traits (e.g. disease resistance, oil quality and components). Due to the availability of the genome sequences of *J. curcas* [31], more SNP markers are being indentified by sequencing the four parents of the two mapping populations using next generation sequencing technologies [32] and bioinformatic tools [33].

In this study, we could not map 29 informative microsatellites and six informative SNPs onto the linkage map. Similar situation has been reported in other studies on linkage mapping [13], [15], [29]. The reasons for this may be genotyping errors, big genetic distances between the unmapped and mapped markers and exhibition of unusual patterns of segregation. Therefore, for constructing a linkage map, slightly more (i.e. 10–20%) polymorphic DNA markers than markers expected to be mapped should be used.

To our surprise, the genotypes of the two parental individuals of *J. curcas* were all homozygous at all 245 microsatellites, whereas the genotypes of the parental individual *J. integerrima* showed some degree of heterozygosity at the 245 microsatellite loci. Similar low genetic variations in *J. curcas* were reported [34], [35]. The reasons for the low genetic variations at microsatellite loci in *J. curcas* warrants to be further studied.

### 2. Mapping families

The variation at DNA level is essential to trace the recombination events [36]. The more DNA sequence variation exists, the easier it is to find polymorphic and informative makers. Therefore we constructed two mapping populations with four parental individuals from two different *Jatropha* species (i.e. *J. curcas* and *J. integerrima*). If a DNA marker is informative only in one mapping population, it can already be used in the map construction. By using two mapping populations, more polymorphic can be mapped to the linkage map as compared to the use of only one population. Since the two mapping populations contained over 300 individuals growing under the same conditions and traits of each tree are being routinely recorded, they can be also used in future mapping of QTL [12] for economically important traits to facilitate genetic improvement of *J. curcas*.

### 3. Linkage map

In this study, we constructed a first-generation linkage map with a total of 506 microsatellite and SNP markers. To our best knowledge, this map is the first one in *Jatropha*. The 506 markers were mapped onto 11 linkage groups, presumably corresponding to 11 chromosome pairs of the *Jatropha* genome [4]. The total length of the map was 1440.9 cM and average marker spacing was 2.8 cM, which is sufficient for genome-wide search for QTL for economically important traits [37]. However, we have noticed that on several linkage groups (i.e. LGs 1, 2, 3, 4 and 8), a few marker spaces were larger than 20 cM. Therefore, more markers should be developed and mapped to increase the density of the linkage map and fill in the gaps in large marker space. We are currently still developing large number of microsatellites and SNP markers using next generation sequencing technologies and bioinformatic tools.

For constructing a high density of linkage map, correct ordering of markers on the whole genome is a difficult task. It is necessary to select appropriate software to accomplish this task. The software CRIMAP may be a good choice for constructing a high density linkage map, as with CRIMAP, large linkage groups are ordered stepwise such that a small number of loci are ordered in each round with respect to the given fixed order constructed in the previous round. Most programs for linkage mapping are applicable only for a single family at a time; the family-specific maps are put together afterwards with some extra work [38]. CRIMAP can be applied to construct a consensus map because it can analyze data over all families simultaneously and improve mapping accuracy.

### 4. Comparative genome analysis

The similarity searches against three model plant species reference genomes showed that 96.8% (215/222) *Jatropha* ESTs containing mapped microsatellites and SNPs were considered to be significantly similar to castor bean genome sequences, 202/222 (91.0%) similar to poplar and 172/222 (77.5%%) to *A. thaliana*. We further generated a comparative map between *J. curcas* and *A. thaliana* using 192 conserved DNA markers. In general, DNA markers mapped on one linkage group hit DNA sequences from several chromosomes *A. thaliana*, suggesting the two genomes shuffled. We discovered that synteny blocks in each of the 11 *Jatropha* linkage groups were syntenic to their counterparts of *Arabidopsis* chromosomes, implying certain colinearity for the syntenic chromosome/linkage pairs. The conserved ESTs identified among *Jatropha*, castor, poplar and *A. thaliana* and the data from the comparative genome analysis should facilitate studies on genome evolution and analysis of structural genome, but more importantly should facilitate functional inference of genes in *Jatropha*. The determination of gene functions is difficult in non-model species including *Jatropha*, thus functional genome analysis will have to rely heavily on the establishment of orthologies from model species. Actually, the genome information of *Arabidopsis* is being utilized in our QTL mapping for oil traits in *Jatropha*. The elucidation of OleI gene involved in oleic acid synthesis in the model species including *Arabidopsis* holds enormous potentials for application in *Jatropha* breeding. These results from comparative mapping between *Arabidopsis* and *Jatropha* are useful for mapping candidate genes contributing to agronomic traits from corresponding regions in *Arabidopsis*. We have mapped a QTL underlying oil composition C18:0 (stearic acid) and C18:1 (oleic acid) on LG5 where harboring *Jatropha* OleI gene (unpublished data). Further comparative mapping has spotted OleI counterpart regions in *Arabidopsis*, which will serve as a resource to generate more markers for further fine mapping and will be beneficial to understand whether the QTL effect is really controlled by OleI gene itself or a gene cluster contributing to oil composition.

Further map based cloning is feasible through fine resolution mapping since the high marker density can shorten chromosome walking, and MAS is effective only when the markers are tightly linked to the gene of interest since crossing-over between the gene and markers dramatically decreases. Mapping more ESTs or gene sequences on the linkage map of *Jatropha* should enhance comparative mapping, thereby transferring genome information from model species to *Jatropha*. In our future work, we will prefer to develop and integrate more and more microsatellites and SNPs into the current linkage map for further gene cloning and MAS.

In conclusion, we established a first-generation genetic linkage map consisting of 506 markers with an average marker density of 2.8 cM/marker. We identified complex syntenic relationships between *Arabidopsis* and *Jatropha* through comparative genomics analysis. The first generation linkage map and comparative map should lay a solid foundation for a variety of future genetic and genomic studies such as QTL mapping of agronomic traits and marker assisted breeding for genetic improvement of *Jatropha*. In the future, more DNA markers, especially SNP markers should be identified and mapped to the linkage map to enable mapping QTL for important traits and to conduct genome-wide association studies to understand the causative mutations for phenotypic variations.

## Supporting Information

Table S1 **Detailed information about DNA markers mapped on the linkage map of** ***Jatropha*** **.**
(XLS)

Table S2 **Detailed results of comparative genome analysis by BlastX.**
(XLS)
